# Effect of age and the *APOE* gene on metabolite concentrations in the posterior cingulate cortex

**DOI:** 10.1016/j.neuroimage.2017.03.031

**Published:** 2017-05-15

**Authors:** Sana Suri, Uzay Emir, Charlotte J. Stagg, Jamie Near, Ralf Mekle, Florian Schubert, Enikő Zsoldos, Abda Mahmood, Archana Singh-Manoux, Mika Kivimäki, Klaus P. Ebmeier, Clare E. Mackay, Nicola Filippini

**Affiliations:** a Department of Psychiatry, University of Oxford, Oxford OX3 7JX, United Kingdom; b Functional Magnetic Resonance Imaging of the Brain Centre, University of Oxford, Oxford OX3 9DU, United Kingdom; c Douglas Mental Health University Institute and Department of Psychiatry, McGill University, Montreal, Canada H4H 1R3; d Physikalisch-Technische Bundesanstalt (PTB), Braunschweig, Berlin, Germany; e Center for Stroke Research, Berlin (CSB), Charité ‐ Universitätsmedizin Berlin, Berlin, Germany; f Centre for Research in Epidemiology and Population Health, INSERM, U1018 Villejuif, France; g Department of Epidemiology and Public Health, University College London, United Kingdom

**Keywords:** Aging, *APOE* gene, Magnetic resonance spectroscopy, Posterior cingulate cortex, Precuneus, Metabolites

## Abstract

Proton magnetic resonance spectroscopy (^1^H-MRS) has provided valuable information about the neurochemical profile of Alzheimer's disease (AD). However, its clinical utility has been limited in part by the lack of consistent information on how metabolite concentrations vary in the normal aging brain and in carriers of apolipoprotein E (*APOE*) ε4, an established risk gene for AD. We quantified metabolites within an 8 cm^3^ voxel within the posterior cingulate cortex (PCC)/precuneus in 30 younger (20–40 years) and 151 cognitively healthy older individuals (60–85 years). All ^1^H-MRS scans were performed at 3 T using the short-echo SPECIAL sequence and analyzed with LCModel. The effect of *APOE* was assessed in a sub-set of 130 volunteers. Older participants had significantly higher myo-inositol and creatine, and significantly lower glutathione and glutamate than younger participants. There was no significant effect of *APOE* or an interaction between *APOE* and age on the metabolite profile. Our data suggest that creatine, a commonly used reference metabolite in ^1^H-MRS studies, does not remain stable across adulthood within this region and therefore may not be a suitable reference in studies involving a broad age-range. Increases in creatine and myo-inositol may reflect age-related glial proliferation; decreases in glutamate and glutathione suggest a decline in synaptic and antioxidant efficiency. Our findings inform longitudinal clinical studies by characterizing age-related metabolite changes in a non-clinical sample.

## Introduction

^1^H magnetic resonance spectroscopy (MRS) is a non-invasive technique used to measure the concentration of brain metabolites *in vivo*. Over the last two decades, it has provided useful diagnostic information about brain tumors (Callot et al., 2008), multiple sclerosis (Narayana, 2005), and a wide range of metabolic disorders (Cecil, 2006). It has also been used to characterize the neurochemical profile of depression (Godlewska et al., 2015), mild cognitive impairment (Tumati et al., 2013), Alzheimer's disease (AD) (Graff-Radford and Kantarci, 2013), and other dementias (Kantarci et al., 2004). Given its noninvasive nature and the increasing availability of 3 T MR scanners, ^1^H-MRS has the potential to evolve into a useful clinical modality for psychiatric and neurodegenerative disorders, but it is still largely considered a research technique (Graff-Radford and Kantarci, 2013, Oz et al., 2014). Several drawbacks have limited its clinical utility: the lack of standardized approaches often yields varying results across studies, and there is inconsistent information on how the concentration of brain metabolites vary during healthy aging. Without knowledge of the latter, it is difficult to conduct longitudinal assessments of patients and disassociate alterations in metabolite levels that may be the result of disease progression from those that accompany the aging process (Haga et al., 2009).

The most widely studied metabolites in the brain are N-acetyl aspartate (NAA), creatine (Cr), choline, and myo-inositol, which serve as surrogate markers of neuronal health, energy metabolism, membrane turnover and glial proliferation respectively (Miller, 1991). Glutathione, glutamine, and neurotransmitters, such as GABA and glutamate can also be quantified (Emir et al., 2011b, Novotny et al., 2003) and have been related to behavioral changes in AD (Jahng et al., 2016, Mandal et al., 2015, Mandal et al., 2012, Saharan and Mandal, 2014). Two literature reviews have highlighted the substantial variability in ^1^H-MRS studies of these metabolites within the healthy aging brain (Haga et al., 2009, Reyngoudt et al., 2012). NAA shows the least consistency across studies; it has been found to decrease (Angelie et al., 2001, Brooks et al., 2001, Driscoll et al., 2003, Gruber et al., 2008, Harada et al., 2001, Lundbom et al., 1999), increase (Charlton et al., 2007, Schuff et al., 1999) and remain unchanged with age (Chang et al., 1996, Leary et al., 2000, Pfefferbaum et al., 1999, Reyngoudt et al., 2012, Saunders et al., 1999), depending on the region of the brain and choice of metabolite quantification technique. Similarly, there have been findings of increases (Angelie et al., 2001, Chang et al., 1996, Chiu et al., 2014, Gruber et al., 2008, Leary et al., 2000, Pfefferbaum et al., 1999) or no changes (Brooks et al., 2001, Charlton et al., 2007, Harada et al., 2001, Reyngoudt et al., 2012, Saunders et al., 1999, Schuff et al., 1999) in the concentration of choline with age across different parts of the brain including both grey and white matter structures. Age-related variations in myo-inositol have been more consistently reported, with most studies showing higher concentrations in older people (Chang et al., 1996, Gruber et al., 2008, Raininko and Mattsson, 2010, Reyngoudt et al., 2012, Ross et al., 2006), although there have also been reports of stable levels of myo-inositol within white matter with age (Leary et al., 2000, Saunders et al., 1999). By comparison, the concentration of glutathione (Emir et al., 2011a) and glutamate (Chang et al., 2009, Grachev and Apkarian, 2001, Kaiser et al., 2005, Marsman et al., 2013, Sailasuta et al., 2008) are known to decline with age across widespread brain regions.

Such discrepancies have been attributed to differences in ^1^H-MRS methods, such as voxel position, data acquisition, analysis and reporting techniques (Haga et al., 2009, Reyngoudt et al., 2012). For instance, reporting metabolite concentrations as relative ratios of creatine (Cr) has been suggested to be misleading (Jansen et al., 2006), as creatine has increasingly been found to vary with age and across different brain regions (Angelie et al., 2001, Chang et al., 1996, Charlton et al., 2007, Chiu et al., 2014, Gruber et al., 2008, Leary et al., 2000, Pfefferbaum et al., 1999, Reyngoudt et al., 2012, Saunders et al., 1999, Schuff et al., 2001). Underpowered studies also contribute to the between-study variability; a systematic review of 18 ^1^H-MRS publications, comparing healthy young (<60 years) and older (>60 years) people, found an average of only about 16 older subjects per study. There is, therefore, an evident need for large-scale studies in healthy older participants (Haga et al., 2009).

Here, we conducted a single voxel ^1^H-MRS study in the posterior cingulate cortex (PCC)/precuneus of 30 younger and 151 older individuals. We focused on this area because it is one of the first regions in the brain to show a decline in structural and functional integrity both during healthy aging as well as in age-related neurodegenerative disorders like AD (Buckner et al., 2005, Greicius et al., 2004, Lehmann et al., 2010). There is considerable evidence for metabolite signatures of late-onset AD (lower NAA/Cr, glutamate, glutamine and glutathione, and higher inositol/Cr) in the cortex of patients relative to healthy controls, particularly within the PCC/precuneus (Graff-Radford and Kantarci, 2013, Kantarci et al., 2013, Kantarci et al., 2007, Kantarci et al., 2004, Kantarci et al., 2002, Kantarci et al., 2000, Mandal et al., 2012, Miller et al., 1993, Riese et al., 2015, Saharan and Mandal, 2014). These metabolic changes may be useful biochemical imaging markers of AD, possibly signifying underlying oxidative, metabolic and neuronal damage. However, relatively little is known about how the metabolite profile of the PCC is affected during normal aging and in people who are at a genetic risk of developing AD (Chiu et al., 2014, Reyngoudt et al., 2012). The *APOE* ε4 allele is the best-established genetic risk factor for sporadic late-onset AD (Bertram and Tanzi, 2009), whereas the rarer (and relatively understudied) ε2 allele is believed to be protective against AD (Suri et al., 2013). There is conflicting information about whether the characteristic metabolite signatures of AD precede its clinical onset and whether or not the *APOE* alleles influence the normal aging process (Gomar et al., 2014, Kantarci et al., 2002, Kantarci et al., 2000). We therefore also investigated the effects of the three *APOE* alleles (ε2, ε3 and ε4) on PCC metabolites.

## Methods and materials

### Participants

The younger and older groups belonged to different studies, each with their own study-specific recruitment protocols (recruitment and genotyping process detailed in Supplementary materials) (Filippini et al., 2014, Suri et al., 2014). Nevertheless, identical scan acquisition protocols, analysis techniques, and exclusion criteria for demographic variables and spectral quality were used for both the age groups. Data from 30 young participants (20–40 years) and 117 cognitively healthy older participants (60–85 years) met the inclusion and exclusion criteria and our strict limits for spectral quality. All older participants were assessed for cognitive impairment using the Montreal Cognitive Assessment (MoCA) and only those with scores ≥26 were included in the study (Nasreddine et al., 2005). *APOE* genotype information was available for all 30 young participants and 100 of the 117 older participants. There were 14 ε2-carriers (n=1 ε2ε2, n=13 ε2ε3), 86 ε3-homozygotes and 30 ε4-carriers (n=2 ε4ε4, n=28 ε3ε4) between 20 and 85 years.

### ^1^H-MRS acquisition and analysis

All participants were scanned at the Oxford Centre for Functional Magnetic Resonance Imaging of the Brain (FMRIB) using a 3 T Verio scanner (Siemens Healthcare, Erlangen, Germany) with a 32-channel head coil. The neuroimaging protocol included:

#### Structural MRI

High-resolution 3D T1-weighted images were acquired using a multi-echo MPRAGE (ME-MPRAGE) sequence (van der Kouwe et al., 2008) (TR=2530 ms, TE=1.79/3.65/5.51/7.37 ms, voxel dimension=1 mm^3^). FMRIB's automated segmentation tool (FAST) was used to segment the structural brain images into grey matter (GM), white matter (WM), and cerebrospinal fluid (CSF) in order to compute the tissue composition of the voxel (Zhang et al., 2001).

#### Single-voxel ^1^H-MRS

**Fig. 1:**
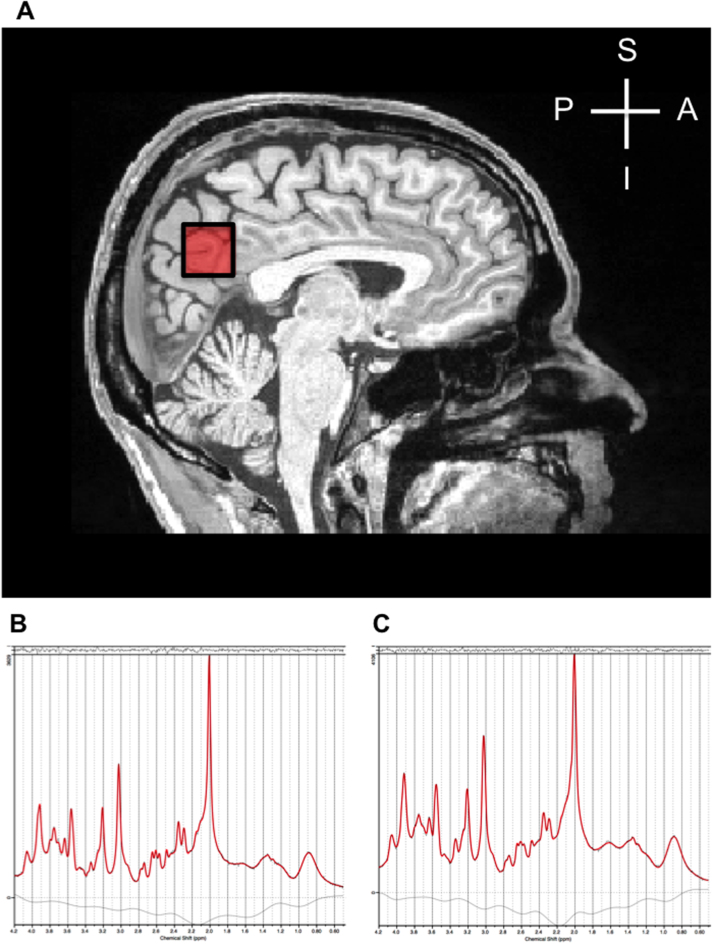
(A) Placement of the 8 cm^3 1^H-MRS voxel in the PCC/precuneus on a midline T1-weighted image. (B-C) Example ^1^H-MRS spectra showing the LCModel fit (Version 6.3-1B) for a participant from the (B) younger and (C) older age group. The lower black curve is the baseline. The data is plotted as a thin black spectrum and the LCModel fit to the data is the thick red spectrum. The top panel contains the residuals, which are fairly scattered about zero, indicating a good fit of the model to the data. Abbreviations: A: anterior, P: posterior, S: superior, I: inferior.

Data were acquired from a 2×2×2 cm^3^ voxel located in the PCC and precuneus region (Fig. 1) similar to the voxel placement in previous studies (Gomar et al., 2014, Kantarci et al., 2004, Kantarci et al., 2000). We positioned the voxel manually by referring to anatomical landmarks on the structural scan, and acquired single volume data at short echo time (TE) using the SPin-ECho full Intensity Acquired Localized (SPECIAL) sequence (Mekle et al., 2009, Near et al., 2013) with Variable Power radio-frequency pulses with Optimized Relaxation delays (VAPOR) water suppression (Tkác et al., 2001) (TE=8.5 s, TR=4000 ms, spectral width=2000 Hz, 128 averages, acquisition time=9 min 6 s). This sequence allows for the simultaneous quantification of several metabolites within a single acquisition without the need for spectral editing, and minimizes signal decay from T2 relaxation; and the reliability and specificity of short-TE ^1^H-MRS measurements of metabolites like glutathione has been described (Deelchand et al., 2016, Godlewska et al., 2015, Mekle et al., 2009, Near et al., 2013, Terhune et al., 2015, Wijtenburg et al., 2014). We used an automated shim tool and outer volume suppression before each scan to saturate spins on all six sides of the voxel. Eight averages of water un-suppressed data were acquired with the same outer volume suppression scheme just prior to the water-suppressed acquisition (Near et al., 2013).

For all spectra, an in-house MATLAB (Natick MA, USA) based semi-automated processing protocol was applied as outlined previously (Near et al., 2013). Briefly, the processing chain involved correction for eddy currents, the removal of motion-corrupted averages and corrections for frequency drifts prior to signal averaging. All processed data were analyzed with LCModel (Version 6.3-1B) (Provencher, 2001), using a basis set that consisted of 21 simulated basis spectra. Macromolecule spectra acquired using an inversion recovery sequence from the anterior cingulate cortex of five independent volunteers (TR=3 s, TI=0.820 s, TE=8.5 ms) were included in the basis set (Kühn et al., 2016). No baseline correction, zero filling or line broadening was applied to the *in vivo* data before input into LCModel. The spectra were fitted over the 0.5–4.2 ppm range. Spectral quality was assessed using strict quality limits (Jansen et al., 2006, Kreis, 2004). The spectra of 34 out of 151 subjects from the older group that did not meet the guidelines were removed from further analysis. All included spectra had Full Width Half Maximum (FWHM)<0.06 ppm (~8 Hz) and signal-to-noise ratio (SNR)>50 as output by LCModel, with no outliers for either of the measures. As reliability criterion we used a Cramer-Rao lower bound threshold of 20% (Jansen et al., 2006, Kreis, 2004). Accordingly, alanine, GABA and lactate could not be reliably measured in over 50% of the participants in the older group and were excluded from further analysis. We measured the water-scaled concentrations of aspartate (Asp), glycerophosphocholine (GPC), phosphocholine (PCh), creatine, phosphocreatine (PCr), glucose (Glc), taurine (Tau), glutamine (Gln), glutamate (Glu), glutathione (GSH), myo-inositol (MI), NAA, N-acetylaspartylglutamate (NAAG). GPC and PCh were combined to quantify total choline content (tCho), and similar measures were made for total creatine (Cr+PCr), total NAA (NAA+NAAG) and total glucose (Glc+Tau), as previously described (Mekle et al., 2009). In agreement with previous reports, we used creatine as a metabolite of interest, rather than as a reference for other metabolites in this study (Jansen et al., 2006). Sample spectra together with their fits from LCModel are presented in Fig. 1.

As expected, the older group had generally broader line widths than the younger group. The two groups belonged to different samples, each with their own study protocols, and the ^1^H-MRS sequences, although identical, were acquired at different times during the multimodal scan protocol. Whereas in the young group the ^1^H-MRS data were acquired towards the start of the protocol, the older group had the ^1^H-MRS sequence towards the end, immediately after high-duty-cycle gradient switching multiband resting-state fMRI [TR/TE=1.3 s/40 ms, field of view=212 mm, 460 volumes, acquisition time=10 min 10 s] and EPI-based DTI acquisition [TR/TE=8.9 s/91.2 ms, field of view=192 mm, b-value=1500 s/mm^2^, 60 directions+ 5 B0, acquisition time=9 min 56 s] (Filippini et al., 2014). The fMRI and DTI acquisitions generated gradient-induced frequency drift which impaired the spectral quantification (Lange et al., 2011). Thus, in order to minimize the effect of longitudinal drift in scanner hardware on the spectra for the older group, we included line width as a confounding covariate in all our analyses and performed an L2 normalisation of the signal intensity of metabolites measured by LCModel. Spectra were normalized to account for the entire metabolic profile, *i.e.* the water-scaled signal intensity of each metabolite was divided by the sum of the water-scaled signal intensities of all metabolites reliably quantified by LCModel (Andronesi et al., 2012). Accordingly, for each subject:

Metabolite_normalized_=Metabolite_raw_/(tCho_raw_+tCr_raw_+tNAA_raw_+MI_raw_+Gln_raw_+Glu_raw_+GSH_raw_+Asp_raw_+tGlc_raw_). Here, “raw” refers to the water-scaled signal intensity of each metabolite.

### Statistical analysis

We used SPSS (SPSS, Chicago IL) for statistical analysis. Sociodemographic variables (age, education), spectral line width, and voxel tissue composition were compared using unpaired *t*-tests (between the two age groups) or one-way analysis of variance (ANOVA) (across the three *APOE* groups). Exact Fisher's test was used for categorical variables (sex). Χ^2^-test was used to confirm that the *APOE* distribution of our sample reflected that expected in a healthy Caucasian population. The effects of age, *APOE*, and the interaction of *APOE*×age on ^1^H-MRS metabolite ratios were computed using a multivariate analysis of covariance (MANCOVA) with *post-hoc* Bonferroni correction for multiple comparisons across the three *APOE* groups.

## Results

### Effect of age on metabolite concentrations

Thirty young and 117 healthy elderly individuals were compared for metabolite concentrations in the PCC/precuneus. The two groups did not differ in years of education or voxel content of white matter (WM) ( Table 1). There were significant group differences in sex, spectral line width, and GM and CSF concentrations within the voxel, and these variables were therefore included as covariates in the model for studying the effect of age on voxel metabolite content.

**Table 1:** Effect of age. Sociodemographics, tissue content and metabolite concentrations within the voxel for the younger and older groups. Values represent means±standard deviations. P values indicate results of chi-square test (for sex), *t*-tests (for sociodemographics, voxel characteristics) and MANCOVA (for metabolite concentrations). Sex, GM% , CSF% and FWHM were included as covariates. ** indicates comparisons which survived *post-hoc* Bonferroni correction for multiple comparisons across metabolites.

	Younger	Older	P
	N=30	N=117	
**Sociodemographics**			
Age (yrs)	23.87±4.95	68.88±5.32	<0.001
Education (yrs)	17.08±2.63	16.01±3.29	0.10
Sex (% males)	56.7%	78.6%	<0.05
**Voxel characteristics**			
GM (%)	0.60±0.03	0.52±0.10	<0.001
WM (%)	0.23±0.03	0.23±0.04	0.72
CSF (%)	0.17±0.03	0.23±0.06	<0.001
FWHM (ppm)	0.026±0.005	0.034±0.006	<0.001
**Neuropsychological tests**			
CES-D	–	3.71±3.98 (range 0–16)	
MOCA	–	28.35±1.15 (range 26–30)	
**Metabolites**			
Aspartate	0.0631±0.004	0.0636±0.006	0.41
Glutamine	0.0447±0.006	0.0538±0.009	0.21
Glutamate	0.2152±0.007	0.1962±0.009	<0.001**
Glutathione	0.0241±0.001	0.0223±0.002	**<0.05**
Myo-inositol	0.1296±0.007	0.1375±0.010	**<0.05**
Total NAA	0.2489±0.007	0.2405±0.012	0.29
Total creatine	0.1732±0.007	0.1810±0.008	<0.001**
Total choline	0.0266±0.002	0.0273±0.003	0.13
Total glucose	0.0747±0.007	0.0779±0.014	0.61
Myo-inosital/Creatine	0.7491±0.049	0.7608±0.061	**0.28**
NAA/Creatine	1.4398±0.078	1.3313±0.093	<0.001*
Myo-inositol/NAA	0.0904±0009	0.1040±0.012	<0.001*

**Fig. 2:**
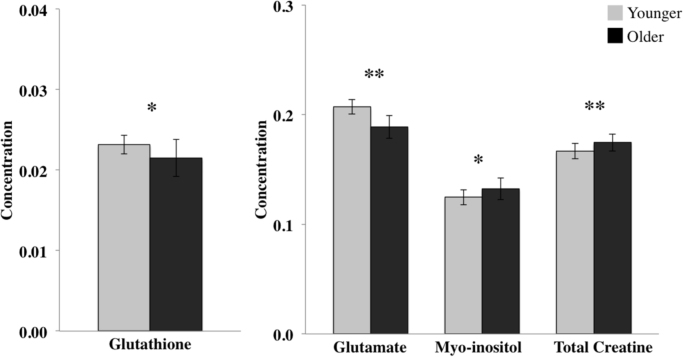
The effect of age on metabolites. Graphs show significant reductions in glutathione and glutamate, and increases in myo-inositol and creatine in the older group. Spectral line width, sex, voxel GM and CSF content were included as covariates. Metabolites are expressed as normalised concentrations. Bars represent means±SD. *p<0.05, **p<0.005.

The MANCOVA revealed a significant effect of age on metabolite concentrations (F_(8,134)_=11.45, p<0.001; Wilk's λ, partial η^2^=0.41). *Post-hoc* comparisons showed that relative to the young group, the older group had significantly higher levels of myo-inositol (F_(1,141)_=5.08, p<0.05; partial η^2^=0.04) and total creatine (F_(1,141)_=33.58, p<0.001; partial η^2^=0.19), and significantly lower levels of glutamate (F_(1,141)_=34.40, p<0.001; partial η^2^=0.20) and glutathione (F_(1,141)_=5.56, p<0.05; partial η^2^=0.04) (Fig. 2). To account for multiple comparisons across the metabolites, we performed an additional Bonferroni correction and accepted statistical significance at p<0.005. Accordingly, only age-related changes in total creatine and glutamate survived. There were no group differences in any other metabolite. For reference, we have also included myo-inositol/creatine, NAA/creatine and myo-inositol/NAA ratios in Table 1. There was a significant decrease in NAA/creatine and increase in myo-inositol/NAA in the older group (p<0.001).

### Effect of APOE on metabolite concentrations

*APOE* information was available for 30 young and 100 of the 117 older participants. Thus, 14 ε2-carriers, 86 ε3-homozygotes, and 30 ε4-carriers aged 20–85 years were compared for effects of *APOE* within the voxel. There was no significant difference in the proportion of young subjects in the three *APOE* groups (χ^2^=0.68, df=2, p=0.71). Further, there were no significant *APOE*-differences in sex (χ^2^=4.42, df=2, p=0.11), age, years of education, spectral line width, voxel CSF, GM or WM content. The multivariate analysis revealed no significant effect of *APOE* (F_(16,234)_=0.68, p=0.81; Wilk's λ, partial η^2^=0.05) or interaction between *APOE* and age group (F_(16,234)_=0.77, p=0.72, Wilk's λ, partial η^2^=0.05) on metabolite concentrations (Table 2). For reference, we have also displayed myo-inositol/creatine, NAA/creatine and myo-inositol/NAA ratios, which did not differ significantly between *APOE* groups.

**Table 2:** Effect of *APOE*. Sociodemographics, tissue content and metabolite concentrations within the voxel for the three *APOE* groups. Values represent means±standard deviations. P values are results of chi-square (for sex), one-way ANOVA (for sociodemographics, voxel characteristics, metabolites).

	ε2-carriers	ε3-homozygotes N=86	ε4-carriers	P
	N=14		N=30	
**Sociodemographics**				
Age (yrs)	55.60±19.57	59.93±19.69	56.26±20.68	0.57
% from older group	71.4%	79.1%	73.3%	0.71
Education (yrs)	17.36±2.93	15.91±3.19	16.40±3.51	0.28
Sex (% males)	78.6%	67.4%	86.7%	0.11
**Voxel characteristics**				
GM (%)	0.53±0.05	0.54±0.10	0.55±0.05	0.77
WM (%)	0.25±0.06	0.23±0.04	0.24±0.04	0.28
CSF (%)	0.22±0.07	0.22±0.06	0.21±0.06	0.88
FWHM (ppm)	0.03±0.01	0.03±0.01	0.03±0.01	0.65
**Metabolites**				
Aspartate	0.0641±0.006	0.0635±0.005	0.0636±0.007	0.92
Glutamine	0.0491±0.008	0.0517±0.010	0.0524±0.007	0.53
Glutamate	0.2013±0.014	0.2002±0.012	0.1997±0.011	0.91
Glutathione	0.0226±0.003	0.0227±0.002	0.0230±0.002	0.81
Myo-inositol	0.1382±0.014	0.1345±0.010	0.1367±0.010	0.33
Total NAA	0.2450±0.013	0.2435±0.012	0.2403±0.011	0.35
Total creatine	0.1757±0.008	0.1799±0.009	0.1801±0.009	0.21
Total choline	0.0273±0.003	0.0271±0.003	0.0272±0.002	0.95
Total glucose	0.0766±0.010	0.0769±0.013	0.0772±0.012	0.99
Myo-inosital/Creatine	0.7861±0.062	0.7487±0.061	0.7595±0.054	0.09
NAA/Creatine	1.3988±0.123	1.3564±0.093	1.3390±0.113	0.19
Myo-inositol/NAA	0.1001±0.017	0.0998±0.012	0.1031±0.014	0.51

## Discussion

To our knowledge this is the first examination of metabolite concentrations in a relatively large sample of cognitively healthy individuals older than 60 years. We have shown age-related alterations in the concentrations of metabolites that are independent of *APOE* genotype. Most importantly, we found that creatine, which has widely been used as a reference metabolite, does not remain stable with age within the PCC/precuneus and may therefore not be a suitable reference in studies involving a broad age-range.

### Effect of age on metabolite concentrations

We compared 30 young and 117 older participants and found significant higher concentrations of total creatine and myo-inositol and lower levels of glutamate and glutathione within the PCC/precuneus of older individuals. Group differences in total creatine and glutamate survived an additional conservative correction for multiple comparisons across metabolites. These changes may point towards alterations in cellular efficiency that are characteristic of the normal aging process.

Our finding of a significant age-related increase in total creatine is supported by several previous reports both within this brain region (Chiu et al., 2014, Reyngoudt et al., 2012) and in other parts of the brain including frontal and parietal grey and white matter (Angelie et al., 2001, Chang et al., 1996, Charlton et al., 2007, Gruber et al., 2008, Leary et al., 2000, Pfefferbaum et al., 1999, Saunders et al., 1999, Schuff et al., 2001), although stable levels of creatine have also been noted in the frontal lobe of males (Brooks et al., 2001). Creatine is a marker of energy metabolism and it is predominantly found in glia (Urenjak et al., 1993). Increases in creatine could be indicative of age-related glial proliferation (Charlton et al., 2007, Leary et al., 2000, Reyngoudt et al., 2012), particularly when observed together with a rise in the concentration of another prominent glial marker, myo-inositol (Brand et al., 1993), as is the case in our study and other investigations (Chang et al., 1996, Gomar et al., 2014, Gruber et al., 2008, Raininko and Mattsson, 2010, Reyngoudt et al., 2012, Saunders et al., 1999).

Importantly, our findings add to the growing body of evidence expressing reservations about using creatine as a reference metabolite in ^1^H-MRS evaluations of metabolite concentrations, particularly across a wide age-range (Jansen et al., 2006). This is clearly illustrated in our comparison of the water-scaled NAA and myo-inositol concentrations that were normalized to the summated metabolites with those normalized to creatine. NAA is perhaps the most widely studied metabolite in the brain. It is present exclusively in neurons (Urenjak et al., 1993). Levels of NAA are altered in neurodegenerative diseases (Graff-Radford and Kantarci, 2013), but reports within the healthy aging brain have been far less consistent, with studies finding increases, decreases and no change in NAA/Cr (Haga et al., 2009, Reyngoudt et al., 2012). However, while observed decreases in NAA/Cr have been interpreted as decreases in levels of NAA, they may in fact simply be methodological artefacts that are wholly explained by increases in creatine (Haga et al., 2009, Reyngoudt et al., 2012). Our observation of no significant age-related change in normalized water-scaled NAA concentrations, but a significant decline in NAA/Cr, supports this notion that NAA/Cr studied over a wide age-range may be confounded by an age-related increase in creatine. In agreement with our findings, previous cross-sectional (Gomar et al., 2014, Reyngoudt et al., 2012) and longitudinal reports (Kantarci et al., 2007, Schott et al., 2010) have found that PCC NAA concentrations remain stable with age, with a recent study concluding that whole-brain NAA is also conserved during normal aging (Wu et al., 2012). Stable PCC NAA levels may suggest a lack of measurable age-related loss in number of neurons within the PCC, but it does not rule out the existence of neuronal shrinkage in this region (Reyngoudt et al., 2012), the latter also being a prominent feature of the aging brain.

We also found significantly lower levels of glutathione and glutamate in the older age group. Glutathione is an antioxidant that regulates the elimination of toxic oxidative stressors (Forman et al., 2009) and has been reported to decrease with age (Emir et al., 2011b). This decline may stem from an increase in cellular glutathione consumption because of higher occurrences of reactive oxidative species with age. Alternatively, it may be suggestive of a shortfall in glutathione production, which could eventually lead to downstream deficits in protection from oxidative stress (Maher, 2005). Both ideas lend support to the free radical theory of aging (Beckman and Ames, 1998). In line with our findings, age-related reductions in glutamate have also been observed in ^1^H-MRS studies of cortical grey matter (Chang et al., 2009, Grachev and Apkarian, 2001, Kaiser et al., 2005, Marsman et al., 2013, Raininko and Mattsson, 2010, Sailasuta et al., 2008). Glutamate is the main excitatory neurotransmitter in the brain, mediating key cognitive and motor functions that are impaired during the normal aging process (Segovia et al., 2001). Unlike creatine and myo-inositol, glutamate is localized primarily in neurons and its concentration in the brain is an indicator of neuronal integrity (Patel et al., 1982). Glutamate reductions have therefore often been attributed to neuronal loss, shrinkage, or a decline in neurotransmission (Kaiser et al., 2005).

### Effect of APOE on metabolite concentrations

This is the first study to characterize the effects all three *APOE* alleles (14 ε2-carriers, 86 ε3-homozygotes and 30 ε4-carriers) on PCC/precuneus metabolites across a wide age-range. We found no effect of *APOE* or an interaction of *APOE* and age group. Our findings are in line with two previous small studies (with largely overlapping samples) of healthy elderly ε3-homozygotes and ε4-carriers, which also found no significant *APOE*-differences in NAA/Cr, myo-inosital/Cr and NAA/myo-inositol within the PCC (Kantarci et al., 2002, Kantarci et al., 2000). In contrast, a larger study of 89 ε3-homozygotes and 23 ε4-carriers found no effect of *APOE* on NAA/Cr, but significantly higher myo-inositol/Cr and Cho/Cr and in old ε4-carriers relative to ε3-homozygotes within the PCC (Gomar et al., 2014). More recently, Riese and colleagues reported significantly lower NAA/Cr concentrations in ε4 carriers (n=9) relative to non-carriers (n=27), but found no *APOE*-related changes in GABA and glutamate+glutamine (Riese et al., 2015). However, 50% of the ε4 carriers in Riese and colleagues’ study had amnestic mild cognitive impairment, which makes it difficult to disassociate the effects of *APOE* from any interaction between *APOE* and underlying amyloid pathology.

The lack of observable *APOE* effects in our study may be attributed to several factors. We were limited by the constraints of a cross-sectional design and potential population biases. Our older group was screened for cognitive impairment and there is a possibility that we may have only included “survivors” who have sidestepped their genetic status for cognitive decline (Gomar et al., 2014). This may have diminished any potential modulatory effect of the ε4 allele. We also did not have sufficient ε2 and ε4 homozygotes to test for a gene-dose effect. Alternatively, it could be that any potential underlying effects of *APOE* on brain metabolites are region-specific, and that the PCC may not be the ideal region of interest to study *APOE*, particularly in cognitively healthy individuals. Recent studies point towards effects of ε4 on NAA/Cr and myo-inositol/Cr within the hippocampus in healthy individuals and MCI patients (Calderon-Garciduenas et al., 2015, Yin et al., 2015). Although the reproducibility of measurements obtained from ^1^H-MRS of the hippocampus may be lower than in other brain regions (Geurts et al., 2004), advances in acquisition techniques have allowed for more reliable quantification of hippocampal metabolites (Allaili et al., 2015, Bednarik et al., 2015). Given the relevance of the hippocampus in studies of *APOE* and AD, future examinations of the pre-clinical effects of *APOE* should consider focusing on this region. Our findings suggest that *APOE* may not strongly influence PCC/precuneus metabolite concentrations in cognitively healthy individuals and that any gene effects in this region that have been observed in AD patients (*e.g.* lower NAA/Cr, higher myo-inositol/Cr) may relate to later stages of the neuropathological cascade.

### Limitations and conclusions

This study offers an improved understanding of age-related changes in metabolite concentrations. However, we must consider some limitations when interpreting our results. First, metabolite concentrations vary between different parts of the brain and between grey and white matter (Angelie et al., 2001, Kreis, 2004, Schuff et al., 2001). Age-dependent changes are therefore likely to be specific to the examined brain region, and our findings must be interpreted in the context of the PCC/precuneus. Second, because of the increasing speculation about the use of creatine as a reference, particularly in studies of subjects with a wide age-range, we refrained from reporting metabolites referenced to creatine in this study. We have therefore reported normalized values of metabolites for each subject, which were referenced to the sum of all reliably estimated metabolites within the spectrum. Although this approach is unconventional, it has been used previously and it seems unlikely that it would introduce systematic biases (Andronesi et al., 2012). Third, metabolite levels are known to vary in neurodegenerative disorders like AD (Graff-Radford and Kantarci, 2013, Kantarci et al., 2002, Kantarci et al., 2000) and without longitudinal follow-up we cannot entirely rule out the possibility of participants from the older group being in the early stages of dementia. However, since we employed a cognitive screening test for this group, it is unlikely that the observed age-related changes in metabolites reflect neurodegenerative processes and are, instead, more suggestive of neurological changes accompanying normal aging. Fourth, our study did not include participants who were 40–60 years old and future studies should consider focusing on this important age range so as to obtain a more complete understanding of the metabolite profile during aging.

We have addressed the current lack of consistent information on age-related changes in the concentration of metabolites and the need for large-scale studies of older populations by examining ^1^H-MRS measurements in a relatively large sample of cognitively healthy individuals. We focused on a single voxel within the PCC/precuneus, which is a region of interest in studies of neurodegeneration but has thus far been understudied in the context of healthy aging. We have shown that metabolites within the PCC/precuneus are susceptible to the aging process and our findings can better inform studies involving longitudinal patient follow-up, where changes in metabolite levels resulting from disease progression may be confounded by those secondary to normal aging.

## Funding sources

Collection of blood and buccal mucosal samples for the older group was supported by the UK Medical Research Council Grant K013351 and the ESRC professional fellowship scheme to Kivimäki. Work on the Whitehall II study was mainly funded by the “Lifelong Health and Wellbeing” Programme Grant: “Predicting MRI abnormalities with longitudinal data of the Whitehall II Substudy” (UK Medical Research Council: G1001354). SS was funded by the University of Oxford Clarendon Scholarship. NF and AM are funded by the HDH Wills 1965 Charitable Trust (Nr: 1117747). ASM receives research support from the US National Institutes of Health (R01AG013196, R01AG034454). CJS holds a Sir Henry Dale Fellowship jointly funded by the Wellcome Trust and the Royal Society (Grant Number 102584/Z/13/Z). UE is funded by the Wellcome Trust, Grant no. 097813/Z/11/Z. CEM is supported by the National Institute for Health Research (NIHR) Oxford Biomedical Research Centre based at Oxford University Hospitals NHS Trust and University of Oxford. The views expressed are those of the author(s) and not necessarily those of the NHS, the NIHR or the Department of Health. Authors do not report any conflict of interest.
