# Supplementary material for: Effect of age and the APOE gene on metabolite concentrations in the posterior cingulate cortex
Source: Neuroimage. 2017 May 15;152:509–16. doi: 10.1016/j.neuroimage.2017.03.031 (PMC5440729; doi:10.1016/j.neuroimage.2017.03.031)
Supplement: Supplementary file 1 — Supplementary material [file mmc1.docx]

**Supplementary information**

***Participant recruitment and APOE genotyping***

*Younger group*: Participants in this group belonged to a previously detailed study of 59 subjects recruited based on their *APOE* genotype (Suri et al., 2014). The sample comprised 19 ε2-carriers (ε2ε2/ε2ε3), 20 ε3-homozygotes (ε3ε3) and 20 ε4-carriers (ε3ε4/ε4ε4) aged 20-40 years recruited from Oxfordshire, United Kingdom. Exclusion criteria were current or past neurological or psychiatric disorders, head injury, substance or alcohol abuse, smoking, corticosteroid or diabetic treatment. DNA was extracted from buccal swab samples and genotyped for *APOE* using standard methods. Two ε3-homozygotes were excluded from the analysis: one because of an incidental finding and one whose spectral dataset did not meet the strict guidelines for spectral quality. For our study, 30 subjects (4 ε2-carriers, 18 ε3-homozygotes, 8 ε4-carriers) were randomly selected from this sample so that their *APOE* allele distribution reflected that expected in a healthy Caucasian population (*χ*^2^=1.82, df=5, *p*=0.87) (Menzel et al., 1983) and was similar to the *APOE* distribution in the older group. The study was approved by a local Ethics Committee (10/H606/34), and informed consent was obtained from all participants.

*Older group*: Participants in this group belonged to the Whitehall II (WHII) imaging sub-study (detailed study protocol described elsewhere (Filippini et al., 2014)). One hundred and fifty one subjects from this cohort who received an ^1^H-MRS scan met the inclusion criteria for this study. Ethical approval was granted generically for the “Protocol for non-invasive magnetic resonance investigations in healthy volunteers” (MSD/IDREC/2010/P17.2) by the University of Oxford Central University/Medical Science Division Interdisciplinary Research Ethics Committee (CUREC/MSD-IDREC), who also approved the specific protocol: “Predicting MRI abnormalities with longitudinal data of the Whitehall II sub-study” (MSD-IDREC-C1-2011-71). The Health Research Authority NRES Committee South Central –Oxford B approved the Study: “The Whitehall II Immune Function Sub-study” (REC reference: 13/SC/0072, IRAS project ID: 120516) and informed consent was obtained from the volunteers. All participants were screened for depression using the Centre for Epidemiological Studies Depression scale (CES-D) (Radloff, 1977) and the Structured Clinical Interview for DSM-IV-TR Axis I Disorders (SCID-I) (First et al., 2002) and cognitive impairment, using the Montreal Cognitive Assessment (MoCA) (Nasreddine et al., 2005). Tests were administered by a trained graduate psychologist or psychiatrist. Exclusion criteria were current neurological disorders, incidental MRI findings, CES-D scores > 16 and/or a diagnosis of current or past depression as indicated by the SCID-I, and MoCA scores < 26. The MRS scans of 34 out of the 151 subjects were rejected in keeping with strict guidelines for spectral quality including spectral artefacts, spectral line width and signal-to-noise. Accordingly, a final sample of 117 neurologically and cognitively healthy subjects with high spectral quality was used to study changes in metabolites with healthy aging.

For our analysis of the effects of *APOE*, DNA was extracted from blood samples drawn using Vacutainer CPT tubes (Becton Dickinson) and genotyped for the *APOE* gene. Genotyping was successful for 101 out of the final 117 subjects and the *APOE* distribution reflected that expected in a healthy Caucasian population (*χ*^2^=3.4, df=5, *p*=0.64) (Menzel et al., 1983). Subjects with the ε2ε4 genotype (n=1) were excluded from the analysis. Thus, 100 older subjects (10 ε2-carriers, 68 ε3-homozygotes, 22 ε4-carriers) were included in our analysis of the effects of *APOE* on PCC metabolites.

**References**

Filippini, N., Zsoldos, E., Haapakoski, R., Sexton, C.E., Mahmood, A., Allan, C.L., Topiwala, A., Valkanova, V., Brunner, E.J., Shipley, M.J., Auerbach, E., Moeller, S., Uğurbil, K., Xu, J., Yacoub, E., Andersson, J., Bijsterbosch, J., Clare, S., Griffanti, L., Hess, A.T., Jenkinson, M., Miller, K.L., Salimi-Khorshidi, G., Sotiropoulos, S.N., Voets, N.L., Smith, S.M., Geddes, J.R., Singh-Manoux, A., Mackay, C.E., Kivimäki, M., Ebmeier, K.P., 2014. Study protocol: The Whitehall II imaging sub-study. BMC Psychiatry 14, 159. doi:10.1186/1471-244X-14-159

Menzel, H.J., Kladetzky, R.G., Assmann, G., 1983. Apolipoprotein E polymorphism and coronary artery disease. Arteriosclerosis 3, 310–5.

Nasreddine, Z.S., Phillips, N.A., Bédirian, V., Charbonneau, S., Whitehead, V., Collin, I., Cummings, J.L., Chertkow, H., 2005. The Montreal Cognitive Assessment, MoCA: a brief screening tool for mild cognitive impairment. J. Am. Geriatr. Soc. 53, 695–9. doi:10.1111/j.1532-5415.2005.53221.x

Radloff, L.S., 1977. The CES-D Scale: A Self-Report Depression Scale for Research in the General Population. Appl. Psychol. Meas. 1, 385–401. doi:10.1177/014662167700100306

Suri, S., Mackay, C.E., Kelly, M.E., Germuska, M., Tunbridge, E.M., Frisoni, G.B., Matthews, P.M., Ebmeier, K.P., Bulte, D.P., Filippini, N., 2014. Reduced cerebrovascular reactivity in young adults carrying the APOE ε4 allele. Alzheimers. Dement. doi:10.1016/j.jalz.2014.05.1755
